# Recovery of Olfactory Function in Postviral Olfactory Dysfunction Patients after Acupuncture Treatment

**DOI:** 10.1155/2016/4986034

**Published:** 2016-02-29

**Authors:** Qi Dai, Zhihui Pang, Hongmeng Yu

**Affiliations:** Department of Otolaryngology, Eye, Ear, Nose & Throat Hospital, Shanghai Key Clinical Disciplines of Otorhinolaryngology, Fudan University, 83 Fenyang Road, Shanghai 200031, China

## Abstract

*Introduction*. The aims of this study were to assess the impact of traditional Chinese acupuncture (TCA) in postviral olfactory dysfunction (PVOD) patients who were refractory to standardized treatment and to compare the results with the impact observed in an observation group.* Methods*. Fifty patients who presented to the outpatient clinic with PVOD and were refractory to standardized treatment were included: 25 were treated with TCA and 25 patients were simply observed. A subjective olfactory test was performed using the University of Pennsylvania Smell Identification Test (UPSIT). The effects of TCA were compared with the results obtained in the observation group.* Results*. Improved olfactory function was observed in eleven patients treated with TCA compared with four patients in the observation group. This study revealed significantly improved olfactory function outcomes in patients who underwent acupuncture compared with the observation group. No significant differences in olfaction recovery were found according to age, gender, or duration of disease between the two groups; however, hyposmic patients recovered at a higher rate than anosmic patients.* Conclusion*. TCA may aid the treatment of PVOD patients who are refractory to drugs or other therapies.

## 1. Introduction

Olfactory impairment is a public health concern. Estimates of its prevalence vary widely, from as low as 3.7% to as high as 75%, depending on the subject's age, the tests employed, and the population evaluated [1]. Many investigators report that olfactory impairment affects approximately one-fifth of the general population [2], with the highest prevalence observed in older men, especially in individuals who suffer from degenerative diseases such as Parkinson's disease and Alzheimer's disease [3]. Apart from degenerative diseases, the most common etiologies of olfaction loss are postviral upper respiratory infection (URI) (18–45% of the clinical population), nasal/sinus disease (7–56%), head trauma (8–20%), exposure to toxins/drugs (2–6%), and congenital anosmia (0–4%) [4]. Postviral URI is among the most common etiologies of olfactory dysfunction observed in ear, nose, and throat clinics. Whereas the literature reports that systemic and topical steroids [5], vitamin B supplementation [6], and other drugs have been used to treat postviral olfactory dysfunction (PVOD), many PVOD patients do not recover olfaction after standardized treatments [7].

Traditional Chinese acupuncture (TCA) is one of the oldest healing methods in China; it has been used for at least 2,000 years. Recently, studies published in the Chinese literature have reported that acupuncture may regulate the microcirculation under physiological or pathological conditions [8]. Moreover, a study by Brandt et al. [9] reported significant improvement in smell and taste functioning after acupuncture treatment. One case report noted the effectiveness of acupuncture for treating anosmia [10]. Vent et al. [11] used TCA to treat 15 patients with PVOD; eight of these patients experienced improved olfactory function, compared with only two patients who were treated with vitamin B complex. The current authors believe that TCA potentially offers a new therapeutic option for postviral dysosmia.

The aims of this historical cohort study were to assess the effects of TCA in PVOD patients who did not recover olfaction after drug treatment and to compare the results obtained using these methods with those obtained in an untreated control group.

## 2. Materials and Methods

Between October 2012 and January 2015, 114 patients who had visited the Department of Otolaryngology at the Eye, Ear, Nose and Throat Hospital of Fudan University in Shanghai, China, reported a reduced subjective sense of smell after an upper respiratory tract infection. The inclusion criteria were PVOD that failed to improve or resolve for more than one month after the oral administration of steroids or vitamin B followed by the topical application of steroid drops to the olfactory cleft or olfactory training. The exclusion criteria included all other conditions associated with olfactory dysfunction, such as a history of head trauma, acute or chronic rhinosinusitis, allergic rhinitis, degeneration diseases, or abnormal nasal anatomy. We explained the acupuncture procedures to the patients who were selected for inclusion. The patients who understood and agreed to TCA were assigned to the TCA group (55). Patients who did not accept TCA were assigned to the observation group (59). Because the PVOD of these patients failed to improve or resolve for more than one month under drug or other therapies, we included a control group that was not administered any drugs and simply observed. The study was approved by the Institutional Review Board of the Eye, Ear, Nose and Throat Hospital of Fudan University, Shanghai, China.

A total of 50 patients were eligible to be included in the study. Two groups of 25 patients each were selected at random to form the observation group and the TCA group. The mean patient age was 51 years (±12.5 SD; range, 23–80 years). The patients were thoroughly examined by experienced otorhinolaryngologists using endoscopic investigation of the nasal cavity and a CT scan of the head, if deemed necessary, to exclude nasal pathologies. A thorough medical history based on both Western medicine and traditional Chinese medicine (with special consideration of energy levels) was acquired using standardized questionnaires. The patients reported that their olfactory dysfunction had persisted for a range of 1–96 months without parasomnia.

The acupuncture treatments were performed by trained and experienced Chinese physicians (PZH and YHM). The treatment was supervised by Professor Zhang Chong Hua, who has been involved in traditional Chinese medicine for more than 50 years and is highly experienced with acupuncture, to ensure the safety of the treatment.

The acupoints chosen for the TCA treatments are referred to as yingxiang, shangyingxiang, and biqiu. Yingxiang (International Number LI20) is a member of the LI (Large Intestine Meridian of the Hand-Yangming) and is located in the middle of the nasolabial groove, beside the nasal alae. The International Number of Shangyingxiang is EX-HN8. Extra points were located superior to the nasolabial groove at the junction of the superior alae nasi and the nasal dorsum. Biqiu is located anterior to the middle turbinate, which differs from its anatomical location in Western medicine. The needle may be turned clockwise upon insertion for tonification or counterclockwise for a more sedating effect on the points and energy centers. The needles were left in place for 20 minutes. TCA was administered three times a week for a total of 10 times per course. After each course, the patients rested for 3–5 days. The treatment continued for three months. Olfactory function was evaluated using the University of Pennsylvania Smell Identification Test (UPSIT) (Sensonics, Inc., Haddon Heights, New Jersey, USA) before and after treatment. The sum of the test results was used as a measure of olfactory function, which allowed the grouping of patients into anosmic (score ≤ 15), hyposmic (15 < score ≤ 35), and normosmic (35 < score) groups. The test results of the groups treated with TCA and acupoint injection were compared with the test results of the observation group. Treatment success was defined as a score increase of at least four points.

### 2.1. Statistical Analyses

Statistical analyses were performed using SPSS software. The results are expressed as the means ± SD in the text and tables. The patients' age, gender, disease severity, and duration of disease were obtained at baseline. Pearson's correlation was used to analyze two-variable correlations. *P* < 0.05 was considered statistically significant.

## 3. Results

Between October 2012 and January 2015, 114 PVOD patients who reported a reduced subjective sense of smell visited the Department of Otolaryngology at the Eye, Ear, Nose and Throat Hospital of Fudan University in Shanghai, China. Of these, 50 patients were selected at random for study among those who were eligible. No harmful or adverse events occurred during or after the treatment phases in either of the two treatment groups. The patient characteristics, including age, gender, disease severity, and duration of disease, are summarized in Table 1. No significant differences were found among the groups regarding their baseline results. Disease duration was classified as 1–3 months, 4–6 months, or longer than 6 months; the age groups were classified as 20–40 years, 40–60 years, and 60–80 years.

**Figure 1:**
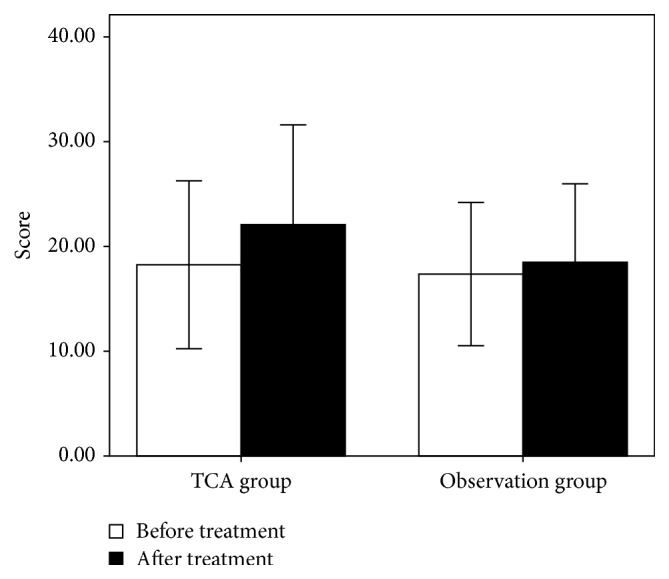
Scores of the two groups before and after treatment. The results are presented as the means ± standard deviation.

Table 2 shows the UPSIT scores for the two groups before and after treatment. The mean score of the TCA group improved from 18.24 ± 8.01 to 22.08 ± 9.52 after acupuncture, and the mean score of the observation group improved from 17.36 ± 6.84 to 18.64 ± 7.70. The pretreatment scores of the two groups were not significantly different, whereas the posttreatment scores of the TCA differed significantly from those of the observation group (Figure 1).

**Figure 2:**
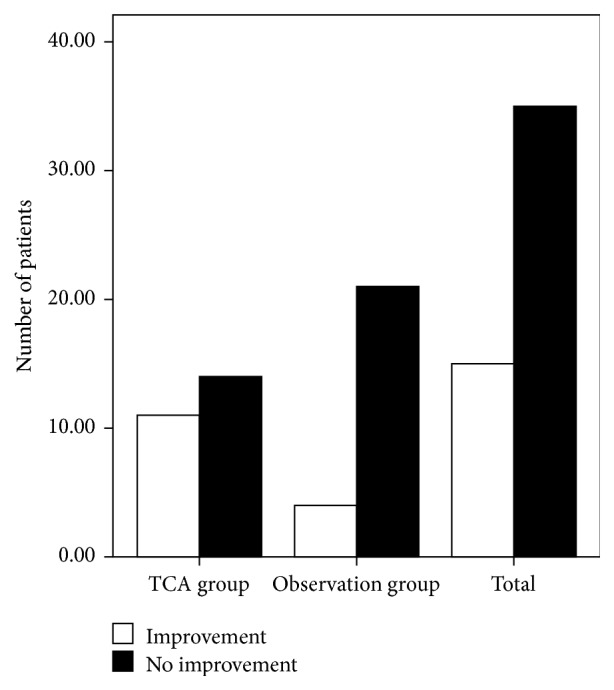
Change in the olfactory function of the two groups.

According to our definition of treatment success (a score increase of at least 4 points), 11 of the 25 patients in the TCA group and 4 of the 25 patients in the observation group showed improved olfactory function (Figure 2). The success rates of the TCA and observation groups were 44.0% and 16.0%, respectively. The Pearson *χ* ^2^ value of the TCA and observation groups was 0.031. These results suggest a significant improvement in the olfactory function outcome of the patients who underwent acupuncture compared with the patients in the observation group.

**Figure 3:**
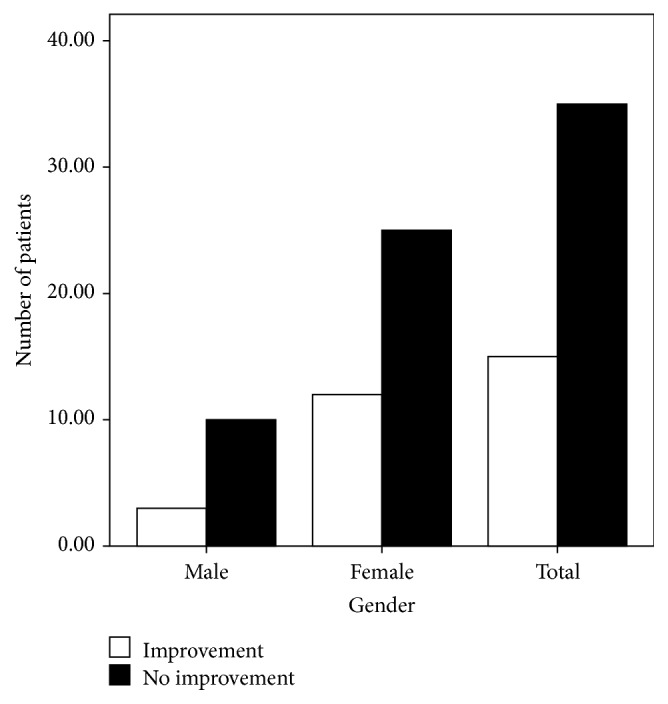
Recovery according to gender.

**Figure 4:**
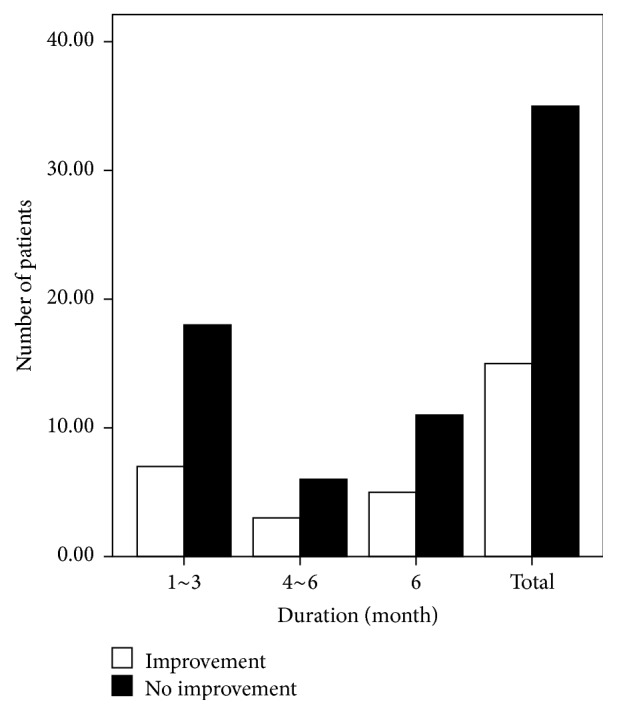
Recovery according to disease duration.

**Figure 5:**
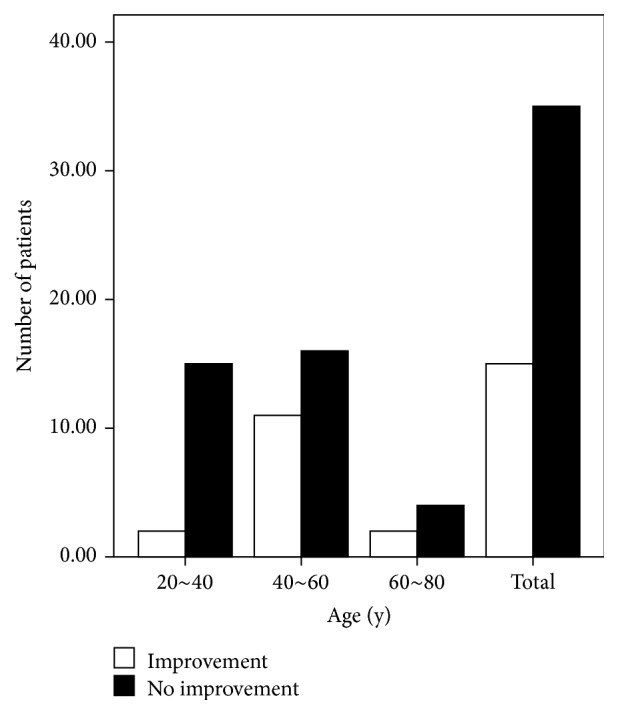
Recovery according to age.

**Figure 6:**
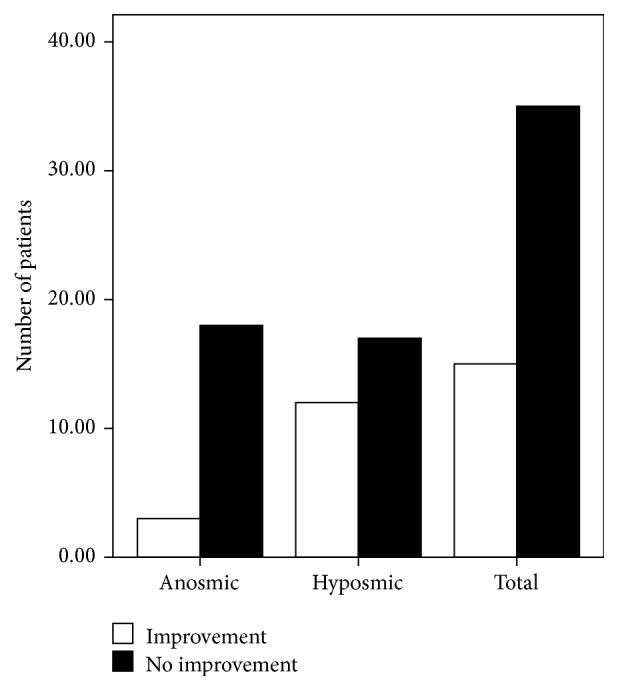
Relationship between disease severity and recovery.

More female than male patients completed the study (37 females versus 13 males). Although three males and 12 females exhibited improved olfactory function after treatment, the factor “gender” had no significant effect on recovery (*P* = 0.527; Figure 3). According to our disease duration classification, of the 25 patients with a disease duration of 1–3 months, seven exhibited improved olfactory function; of the nine patients with a disease duration of 4–6 months, three presented improved olfactory function; and of the 16 patients with a disease duration longer than 6 months, five presented improvement. However, the factor “duration” also had no significant effect on recovery (*P* = 0.948; Figure 4). Similarly, the factor “age” did not affect recovery (*P* = 0.122). Of the 17 patients in the group of patients who were 20–40 years of age, two showed improvement; of the 27 patients in the group of patients who were 40–60 years of age, 11 showed improvement; and of the six patients in the group of patients who were 60–80 years of age, two showed improvement (Figure 5). Twelve of the 29 hyposmic patients and three of the 21 anosmic patients showed improvement. The hyposmic patients recovered at a higher rate than the anosmic patients (*P* = 0.039; Figure 6).

## 4. Discussion

As Table 1 shows, the baseline results did not significantly differ among the groups; therefore, the two groups were suitable for comparison. In this study, approximately half of the patients suffering from PVOD showed improved olfactory function after TCA; however, in the observation group, only four patients improved. TCA has been shown to be useful under many circumstances [8–16]. This study shows that TCA may be useful for the treatment of PVOD in patients who do not present olfactory function improvement after standardized treatments.

Many studies have suggested that olfaction may change and recover; therefore, the possibility of spontaneous recovery should be considered. Hendriks [17] reported a 35% recovery rate over approximately 12 months. Reden et al. [18] reported that 32% of patients exhibited significant improvement after a mean interval of 14 months. Heilmann et al. [6] showed that 35% of patients exhibited a marked increase in olfactory function over an average period of four months. Consistent with these observations [7, 17, 18], our results indicate that approximately one-third (32%) of patients with PVOD exhibit improvement over approximately one year, and longer durations between baseline and posttreatment testing correlated with a higher probability of olfactory function improvement. These data provide valuable information about olfactory disorders, including their natural history and prognosis. As shown in Figure 1, the pretreatment scores of the two groups did not differ significantly; however, the differences among the groups were statistically significant after treatment/observation. Unlike the pretreatment scores, the posttreatment scores of the TCA group differed significantly from those of the observation group. We found that 44% of the patients in the TCA group exhibited a recovery in olfactory function over a 3-month period. This recovery rate is higher than the 32% cited in the literature despite the fact that our observation time was less than one year. This result appears to provide further evidence of the effectiveness of TCA.

We have often observed that females are more susceptible to PVOD than males; in our study, the female-to-male ratio was 5 : 2. However, recent data show that women are more resistant to viral infections due to higher levels of estradiol [19]. Although in our study, recovery was unrelated to gender, which is consistent with the results obtained by Reden et al. [18].

Age was also considered. The effect of aging on olfactory dysfunction has been investigated extensively, and many studies have shown that olfactory function decreases as a function of age [20–22]. For example, Mori et al. [21] report observing PVOD in more than 70% of subjects aged 50 to 69 years. Many investigators believe that olfactory receptor neuron degeneration decreases with age, especially in individuals who suffer from degenerative diseases such as Parkinson's disease and Alzheimer's disease [3], causing an overall decrease in the number of olfactory receptor neurons. This decrease may explain why the nasal mucosa and olfactory epithelium of the elderly are more vulnerable to infections and why the recovery of olfactory function after PVOD decreases with age. In our study, we excluded individuals with degenerative diseases to ensure that they would not influence the results. In our study, 40- to 60-year-old patients comprised 57% of the 114 PVOD cases. Similar to the results obtained by Reden et al. [18], our results showed a negative correlation between age and the recovery of olfactory function.

Disease duration also represents a prognostic factor. As previously reported [21, 22], the likelihood of recovery decreases as the duration of olfactory loss increases. However, recent data have confirmed that the duration of olfactory impairment and changes in olfactory function scores are negatively correlated [18], a finding that was replicated in our study.

Among the 114 patients admitted to the Department of Otolaryngology at the Eye, Ear, Nose and Throat Hospital of Fudan University, 46 were anosmic and 68 were hyposmic. Among those who completed the study, two of the 15 anosmic patients and 13 of the 35 hyposmic patients showed improved olfactory function; this result appears to confirm that hyposmic patients have a better prognosis.

Acupoint has a long history in traditional Chinese medicine. Doctor Wang Zhi-zhong of the Southern Song Dynasty developed the concept of “location of disease,” which refers to the clinical implications of various acupoints. Unlike pharmacologic therapies, the aim of acupuncture is to restore physical balance and to bring yin and yang into equilibrium. Our results are supported by a study on the use of acupuncture in PVOD by Vent et al. [11] wherein 15 PVOD patients were treated with needle acupuncture. In the acupuncture group, eight out of 15 patients improved (based on the authors' definition of treatment success); in comparison, only two of the 15 patients in the control group improved. However, according to Chinese acupuncture guidelines, therapy should be individualized. To conduct a double-blind study, acupuncture and injection placebos would need to be administered to the control group; however, in this study, it was not feasible to use an acupuncture placebo. In Chinese acupuncture, every needle insertion is believed to exert an effect; therefore, the sham insertion of a needle at a random position could have negative effects on the patient's energy balance [11].

This is an important study that investigates the effects of acupuncture in PVOD patients who are refractory to standardized treatments. A key finding of this historical cohort study is that TCA appears to be beneficial in the treatment of PVOD, which provides hope for those who are refractory to drugs or other treatments.

## 5. Conclusion

TCA may aid the treatment of PVOD patients who do not recover olfaction through the use of drugs or other treatments. Age, gender, and disease duration have no effect on the prognosis for olfactory dysfunction recovery; however, hyposmic patients tend to have a better prognosis than those with anosmia.

## Figures and Tables

**Table 1:** General characteristics of the patients by group.

	Age	Gender male/female	Olfactory function anosmic/hyposmic	Course of disease 1~3/4~6/6~months
TCA group	54.2 ± 12.7	25 (11/14)	25 (10/15)	25 (10/5/10)
Observing group	49.1 ± 12.8	25 (5/20)	25 (11/14)	25 (15/5/6)

**Table 2:** UPSIT scores of the TCA and observation groups before and after treatment.

	TCA group	Observation group
Before treatment	18.24 ± 8.01	17.36 ± 6.84
After treatment	22.08 ± 9.52	18.64 ± 7.70
